# Antimicrobial resistance among GLASS priority pathogens from Pakistan: 2006–2018

**DOI:** 10.1186/s12879-021-06795-0

**Published:** 2021-12-07

**Authors:** Dania Khalid Saeed, Joveria Farooqi, Sadia Shakoor, Rumina Hasan

**Affiliations:** 1 Department of Pathology and Laboratory Medicine, The Aga Khan University, Karachi, 74800 Pakistan; 2 Faculty of Infectious and Tropical Diseases, London School of Hygiene and Tropical Medicine, London, UK

**Keywords:** Antimicrobial resistance, GLASS priority pathogens, Pakistan, Antibiogram data

## Abstract

**Background:**

In 2018 Pakistan initiated its national antimicrobial resistance (AMR) surveillance aligned with Global Antimicrobial Surveillance System (GLASS). To complement this surveillance, we conducted a situational analysis of AMR rates among GLASS organisms in the country. Data from published studies and from antibiograms was compared and role of antibiograms as potential contributors to national AMR surveillance explored.

**Methods:**

AMR rates for GLASS specified pathogen/antimicrobials combination from Pakistan were reviewed. Data sources included published studies (2006–2018) providing AMR rates from Pakistan (n = 54) as well as antibiograms (2011–2018) available on the Pakistan Antimicrobial Resistance Network (PARN) website. Resistance rates were categorized as follows: Very low: 0–10%, Low: 11–30%, Moderate: 30–50% and High: > 50%.

**Results:**

Published data from hospital and community/laboratory-based studies report resistance rates of > 50% and 30–50% respectively to 3rd generation cephalosporins, fluoroquinolones and cotrimoxazole amongst *Klebsiella pneumoniae* and *Escherichia coli*. Carbapenem resistance rates amongst these organisms remained below 30%. High (> 50%) resistance was reported in *Acinetobacter* species to aminoglycosides and carbapenems among hospitalized patients. The evolution of ceftriaxone resistant *Salmonella* Typhi and *Shigella* species is reported. The data showed > 50% to fluoroquinolones amongst *Neisseria gonorrhoeae* and the spread of methicillin resistant *Staphylococcus aureus* (< 30%; 2008) to (> 50%; 2010) in hospital settings. Resistance reported in published studies aligned well with antibiogram data. The latter also captured a clear picture of evolution of resistance over the study period.

**Conclusion:**

Both published studies as well antibiograms suggest high rates of AMR in Pakistan. Antibiogram data demonstrating steady increase in AMR highlight its potential role towards supplementing national AMR surveillance efforts particularly in settings where reach of national surveillance may be limited.

**Supplementary Information:**

The online version contains supplementary material available at 10.1186/s12879-021-06795-0.

## Background

Antimicrobial resistance (AMR) recognized as a natural evolutionary process is facilitated by the genomic plasticity of microorganisms [1]. In recent times, this process has been greatly accelerated by the increased exposure of microorganisms to antimicrobial agents. Excessive usage of antimicrobial drugs in human and animal populations as well in agriculture contribute to their increasing availability in the environment placing a strong selective pressure within microorganisms and resulting in the development of antimicrobial resistance [2]. At a global level, the emergence and spread of antimicrobial resistance is enhanced by poor prescribing practices, counterfeit drugs, and poor infection control practices. Travel and trade can contribute to this spread; the spread of NDM-1, from Indian sub-continent region to Europe and United States highlights the global nature of this public health disaster [3]. Reports of high levels of antibacterial resistance [4–7] from hospitals as well as the community [8, 9] in Pakistan, the fifth most populous country globally [10], places an additional burden on an overstressed and under resourced health care system. Circulating mobile genetic elements in bacterial species [5, 7, 11] that can transfer multidrug resistance genes within and between species have also been reported. Further, the detection of extensively drug resistant (XDR) *Salmonella* Typhi from Hyderabad, Pakistan [12] reinforces concerns over the complex interaction between environment and anthropogenic activities in contributing towards emerging AMR.

Hence, following the Global Action Plan to tackle antimicrobial resistance at the 68th World Health Assembly, 2015, Global Antimicrobial Surveillance System (GLASS) was established [13] to collate antimicrobial resistance data at a global level.

Responding to the Global Action Plan and reports of increasing resistance in Pakistan, the National Strategic Framework for Containment of Antimicrobial Resistance, was translated into the National Action Plan of Pakistan for Antimicrobial Resistance and launched in 2018, initiating national AMR surveillance system (PASS) aligned with GLASS in 2018 [9]. To complement PASS, which collects data from 2018 onward, we present an analysis of the available information on AMR in the country from 2006 to 2018 based on literature review of published studies on available antibiograms. Resistance patterns over the course of the study period are presented.

## Methods

### Data sources

The situational analysis was conducted using two methods: literature review of published studies and review of data available on the Pakistan Antimicrobial Resistance Network (PARN) [14] website.

#### Review of published literature

Literature search was performed for studies reporting antimicrobial susceptibility rates of GLASS specified microorganisms (GLASS) [13]: *Klebsiella pneumoniae*, *Escherichia coli*, *Acinetobacter baumannii*, *Salmonella* Typhi, *Shigella* species, *Neisseria gonorrhoeae*, *Staphylococcus aureus* and *Streptococcus pneumoniae* from Pakistan.

#### Search strategy

Studies and reports were identified by searching electronic database Medline (PubMed) peer reviewed literature. Key terms used in the search are presented in Additional file 1.

#### Selection criteria

English language articles published January 01, 2006–January 31, 2018 were included. Systematic reviews, case reports, novel antibacterial therapeutics, articles not supported by quality control methodologies, studies focusing on vaccination program outcomes and in-data-review were excluded.

**Fig. 1:**
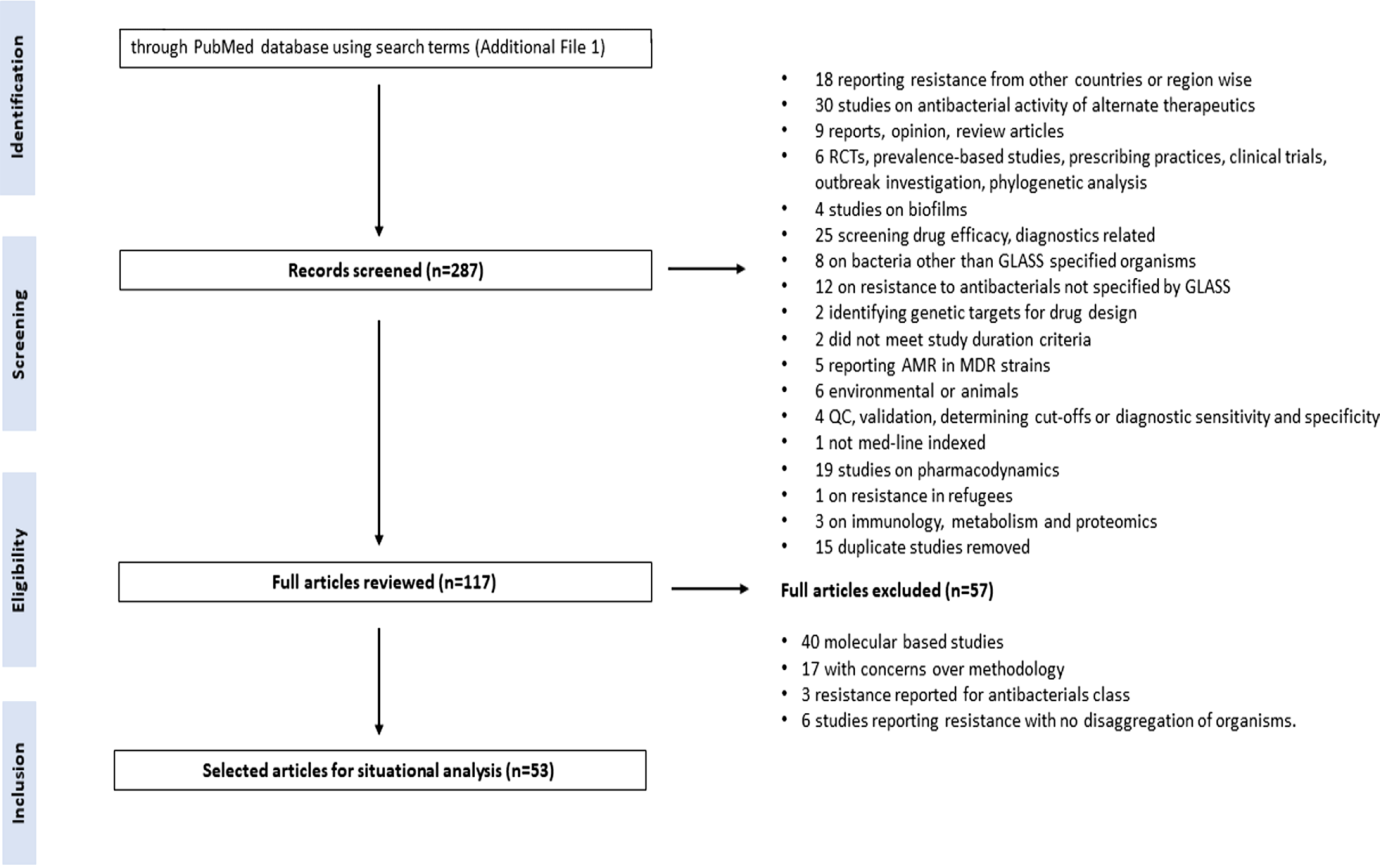
Search methodology. Algorithm showing search methodology and reasons for exclusion; 53 studies selected from a total of 117 studies identified using the search strategies (Additional file 1). *GLASS* Global Antimicrobial Surveillance System, *ARG* antimicrobial resistance genes, *QC* quality control, *RCT* randomized control trial, *MDR* multi drug resistant

Hospital- as well as community-based studies for both adult and paediatric populations were included (Fig. 1).

The papers retrieved were independently reviewed by two authors (DKS and RH). Relevant studies based on selection criteria were included. Discrepancies arising between the reviewers were resolved by consensus discussions between the two reviewers.

#### Outcomes of interest

Data from the reviewed literature was categorized under the following headings in MS Excel: author, journal, year published, year of study, location, organism names, isolate numbers, site of infection, hospital/community, age group, antibiotics tested, percent resistance, multi drug resistance [15], clinical outcomes of treatment based on mortality linked to the infectious episode, methods provided for susceptibility testing, statistical analysis, quality assurance and use of Clinical Laboratory Standards Institute (CLSI) guidelines, key findings and limitations.

#### Antibiogram data

Information on antimicrobial resistance included in antibiograms published on the PARN website was reviewed. To address the gaps and strengthen laboratory capacity for AMR surveillance PARN [14]; a collaborative initiative between public and private health organizations from Pakistan was established in 2006. It is an electronic platform that aims to share data and information relating to antimicrobial resistance in Pakistan. As part of these activities, PARN publishes antibiograms that are contributed voluntarily by individual laboratories. Based on their capacity and interest, contribution by the individual laboratories has varied from year to year. However, cumulatively, such information is considered an effective resource providing Level 2 evidence i.e., laboratory-based information in the absence of standardized AMR surveillance processes [16] for gauging antimicrobial resistance trends in the country. Data from antibiograms available for the years 2011–2018 was included. The antibiogram data available for the study period and included in this analysis were from the following institutions: Aga Khan University (2011–2018), Patel Hospital Karachi (2011–2012), Tabba Hospital Karachi (2011–2012), Indus Hospital Karachi (2011–2012), Dr Ziauddin Hospital (2011), Shifa Hospital Islamabad (2015), Jinnah Hospital Karachi (2014–2018), Civil Hospital Karachi (2016–2018), Shaukat Khanum Memorial Cancer Hospital and Research Centre, Lahore (2017).

#### Data analysis

The pathogens/antimicrobial based on GLASS specified bug/drug combination (GLASS) [13] investigated for resistance from both published reports and from the PARN website are shown in Table 1.

**Table 1:** Global Antimicrobial Resistance Surveillance System (GLASS) specified antimicrobials and bacteria

Organisms	Antibiotics investigated
*K. pneumoniae*	Ceftriaxone/cefotaxime/ceftazidime, imipenem/meropenem, ciprofloxacin/ofloxacin, co-trimoxazole, colistin
*E. coli*	Ampicillin, ceftriaxone/cefotaxime/ceftazidime, imipenem/meropenem, ciprofloxacin, co-trimoxazole, colistin
*Acinetobacter species*	Imipenem/meropenem, gentamicin, amikacin, tigecycline, minocycline
*Salmonella* Typhi	Ceftriaxone, ciprofloxacin
*Shigella* species	Ceftriaxone, ciprofloxacin
*N. gonorrhoeae*	Ceftriaxone, ciprofloxacin, azithromycin, spectinomycin
*S. aureus*	Oxacillin/cefoxitin, vancomycin
*S. pneumoniae*	Penicillin, ampicillin, ceftriaxone, levofloxacin

In order to minimize the effect of heterogeneity arising from different settings in which studies were conducted, the reviewed literature was divided into the following groups:

1. Hospital based studies including Intensive Care Units (ICUs), Medical Intensive Care Units (MICUs), and medical wards.
2. Laboratory based surveillance studies, community studies.
3. Hospital and community-based studies specifically reporting antimicrobial resistance rates in neonates and children.
4. Antimicrobial Resistance data based on published antibiograms.

Resistance rates reported in published studies were included in groups 1–3 while data from antibiograms was included in group 4.

Median percentage resistance along with their confidence intervals were determined for studies/antibiograms published in the same year using STATA/MP 13[17] by calculating the 50th centile resistance rates with 95% CIs. Where susceptibility rates were given, resistance rates were calculated by subtracting the percentage susceptibility rates provided from 100.

Resistance rates were categorized from very low to high as follows:

Very low: 0–10%, Low: 11–30%, Moderate: 30–50% and High: ≥ 50%

## Results

PubMed search returned a total of 287 articles. A total of 53 studies were selected for this review (Fig. 1).

While the majority (40/53) of studies included data from both adults and children, this data was not disaggregated by age (Tables 2, and 3, Additional Files 2 and 3). One study [18] disaggregated data by age and sex but did not report the denominators (entire population sampled). A few studies (n = 12) focused only on the paediatric age group, and data from these are described separately (Table 4, Additional file 4). The majority of the studies included resistance data from hospitalized patients, with only 16 studies reporting community level data. These are included in Table 3.

**Table 2:** Antimicrobial resistance rates (shown as percent resistance) reported in hospital-based studies (2006–2017)

Priority bacteria	Year of publication	Duration of Study	N value	AMP	CRO/CTM	CAZ	MEM/IPM	GEN	AK	CIP/OFX	SXT	TIG	MIN	OXA	VAN	Refs.
*K. pneumoniae*	2009	2006–2007	9		**100**		*0*				**100**					[34]
	2010^α^	2007–2008	72		**62.5**	47.2	20.8			**73.6**	**88.9**					[35]
	2010^α^	2002–2007	15,914				*0.4*			22.4	41.25					[36]
	2016	2015–2016	58		**94**		**56**			**71**	**73**					[19]
	2017	2013–2014	93		**60**		*1.1*				**68.5**					[37]
*E. coli*	2009	2008	29	**86.2**	**82.4**					**75.9**						[38]
	2010	2007–2008	53		75* **(71.7–78.3)**	63.6* (55.5–71.7)	10.1* *(5.1–15.1)*			70.35* **(65.2–75.5)**	**64.2**					[35]
	2010	2009	46													[39]
	2015	2011–2012	166							59.1* **(39.8–78.4)**						[31]
	2015	2012–2013	227	**96.5**	**80.2**		*7*									[40]
	2016	2013–2014	89				10* *(5.6–12)*			60* **(57.3–84)**	60.9* **(61.8–60)**					[41]
	2016	2014	50	95* **(93–98)**	75.5* **(58–93)**											[42]
	2016	2015–2016	87													[19]
	2017	2013–2014	108				2.04* *(1.89–2.2)*			76.7* **(74.04–79.3)**	71.6* **(67.2–76)**					[37]
	2017	2014–2015	351	**90.8**	**78**	45.8										[18]
	2018	2017	119	**71.4**	**65.5**	**64.7**	*5*			**71.4**	**63.9**					[43]
*Acinetobacter* sp.	2009	2009	27				80* **(75–85)**	**92**	**78**							[34]
	2009	2006	4													[44]
	2014	2010–2011	90				59.4* **(53.3–65.5)**		**68.94**			20	**83.3**			[45]
	2014	2010	30													[32]
	2015	2011	26				**96.2**		**50**			11.5				[46]
	2016	2014	51				100* **(17.6–100)**		75* **(37.2–95)**							[42]
	2016	2013–2014	8					98.5* **(97–100)**								[41]
	2016	2015–2016	87													[19]
*S. aureus*	2009	2005–2008	195											21.5	*0*	[27]
	2014	2009–2010	54											48.1* (33.9–63.27)		[29]
	2014	2011	375													[47]
	2014	2011	177													[48]
	2015	–	104											25.97* (2.9–49.04)		[49]
	2015	2013–2014	77													[50]
	2017	2015–2017	346											52* **(26.6.-84.6)**		[28]
	2017	2013–2014	142													[37]
	2017	2013	26													[51]

The pathogen/antimicrobial combination used was in accordance with WHO GLASS. n value: Number of isolates included in the study reported. For *Staphylococcus aureus* in addition to the antimicrobials recommended for reporting in GLASS, vancomycin has also been included

Resistance was graded as: very low (0–10%): in *italic*; low (11–30%): plain font; moderate (30–50%): underlined; high (≥ 50%): **bold font**

*AMP* ampicillin, *CRO/CTM* ceftriaxone/cefotaxime, *CAZ* ceftazidime, *MEM/IPM* meropenem/imipenem, *GEN* gentamicin, *AK* amikacin, *CIP/OFX* ciprofloxacin/ofloxacin, *SXT* sulfamethoxazole and trimethoprim, *TIG* tigecycline, *MIN* minocycline, *OXA* oxacillin, *VAN* vancomycin, *sp*. species

*Where applicable, data from studies conducted during the same year was merged and shown as median percentage (with 95% Confidence Intervals)

^α^Data not merged because of wide study duration

**Table 3:** Antimicrobial resistance rates (shown as percent resistance) from laboratory surveillance and community-based literature review (2009–2018)

Priority bacteria	Year of publication	Duration of study	n	PEN	AMP	CRO/CTM	CAZ	MEM/IPM	AK	CIP/OFX	SXT	AZM	SPT	OXA	VAN	Refs
*K. pneumoniae*	2012	2010–2011	672							34.5						[52]
	2013	2008–2011	1617			26.6	26.7	*1.4*		**53.8**						[53]
	2016	2012–2013	60			21.7										[20]
	2018	2015	59			39		15	*10*	47.5	**51**					[54]
*E. coli*	2012	2010–2011	1290							**58.4**						[52]
	2015	–	20		**90**					**50**	**55**					[55]
	2016	2012–2013	110			*10*										[20]
	2018	2015	188		**97**	**58.5**		*6*		**65**	**70**					[54]
*S. Typhi*	2012	2008–2010	131			*1.3*				*1.31*						[21]
	2014	2009–2011	2576			*0.08*				**88.2**						[22]
	2016	2012–2013	40			*7.5*										[20]
	2017	2014–2015	270			*0*				**93**						[56]
	2018	2012–2014	1979			*0.2*				**90**						[57]
*Shigella*	2009	2006	115			5* *(2–8)*				7.6* *(4.3–10.9)*	78.6* **(73.9–83.2)**					[25]
	2009	2007	101													[25]
	2016	2011–2013	45			*7*				25	**79**					[26]
*N. gonorrhoeae*	2011	2007–2009	112							93.8* **(89.6–98)**						[58]
	2011	2007–2009	318													[59]
	2013	2008–2011	18			*0*				**93.8**		*7.7*	*0*			[60]
	2016	2012–2014	100			*0*				**86**		*1*	*0*			[61]
*S. aureus*	2009	2005–2008	42											11.9	*0*	[27]
	2014	2009–2010	144											27.8		[29]
*S. pneumoniae*	2016	2013–2014	42	**88.2**		35.7					**76.2**					[30]

The pathogen/antimicrobial combination used was in accordance with WHO GLASS. For *Staphylococcus aureus* in addition to the antimicrobials recommended for reporting in GLASS, vancomycin has also been included. n value: Number of isolates included in the study reported

Resistance was graded as: very low (0–10%): in *italic*; low (11–30%): plain font; moderate (30–50%): underlined; high (≥ 50%): **bold font**

*PEN* penicillin, *AMP* ampicillin, *CRO/CTM* ceftriaxone/cefotaxime, *CAZ* ceftazidime, *MEM/IPM* meropenem/imipenem, *AK* amikacin, *CIP/OFX* ciprofloxacin/ofloxacin, *SXT* sulfamethoxazole and trimethoprim, *AZM* azithromycin, *SPT* spectinomycin, *OXA* oxacillin, *VAN* vancomycin, *Sp* species

*Where applicable data from studies conducted during the same year was merged and shown as median percentage (with 95% confidence intervals)

**Table 4:** Antimicrobial resistance rates (shown as percent resistance) amongst pediatric population based on literature review (2010–2017)

Priority pathogens	Year of publication	Year of study	Category	N	AMP	CRO/CTM	CAZ	MEM/IPM	GEN	AK	CIP/OFX	SXT	CLOX	VAN	Refs.
*K. pneumoniae*	2013	2006–2011	I	104				20			20				[62]
*E. coli*	2010**	2009–2010	I	30	**73.3**	**63.4**	**50.3**	*2.9*			40.3				[63]
	2012**	2009–2010	I	30	**73.3**	46	46.2	20			40.3				[64]
	2016	2012–2015	I	811	84.9* **(89.8–80)**	**78**		*5.3*			50.6* **(32.7–68.57)**				[65]
	2016	2010–2011	I	35								77.5* **(77.1–78)**			[66]
	2016	2010–2012	II	46	**91**				40		39				[67]
*Acinetobacter *sp*.*	2012	2009–2010	I	17				**52.9**	**66.6**	46.6					[64]
	2014	2010–2011	I	12				**100**	**100**	**100**					[33]
	2016	2014	I	112				**66**	**71.2**	**83.3**					[68]
	2017	2014	I	100				**94.7**	**100**	**95.78**					[69]
*S. Typhi*	2012^α^	2002–2004	II	189		*0*					*0*				[70]
	2010^α^	2007–2008	II	16		*0*					46				[71]
*S. aureus*	2011	2004–2007	II	304									*4.3*		[72]
	2012	2009–2010	I	35										*0*	[64]

The pathogen/antimicrobial combination used was in accordance with WHO GLASS. n value: Number of isolates included in the study reported. Category of studies included: I: Hospital based studies, II: Lab-based surveillance/Community studies. Data from lab-based (Category I) and community surveillance (Category II) studies were not merged

Resistance was graded as: very low (0–10%): in *italic*; low (11–30%): plain font; moderate (30–50%): underlined; high (≥ 50%): **bold font**

*AMP* ampicillin, *CRO/CTM* ceftriaxone/cefotaxime, *CAZ* ceftazidime, *MEM/IPM* meropenem/imipenem, *GEN* gentamicin, *AK* amikacin, *CIP/OFX* ciprofloxacin/ofloxacin, *SXT* sulfamethoxazole and trimethoprim, *CLOX* cloxacillin, *VAN* vancomycin, *sp.* species

*Data from studies conducted during the same year was merged and shown as median percentage (with 95% confidence intervals)

**Both these studies were from the same institute and thus data overlap likely

^α^The years that these studies were conducted have no overlap, data presented according to the year of study to reflect temporal resistance trends

Antibiogram data available were not further classified by the setting (hospital vs community), site of infection, or age. However, overall resistance rates presented in the antibiograms (Table 5, Additional file 5) for *K. pneumoniae*, *E. coli* and *Acinetobacter* species, *Shigella* species, *N. gonorrhoeae* as well as *S. aureus* approached those in reported studies (Tables 2, 3 and 4).

**Table 5:** Antimicrobial resistance rates from laboratory based antibiograms (2006–2018)

Priority bacteria	Year	PEN	AMP	CRO/CTM	MEM/IPM	GEN	AK	CIP	SXT	OXA	VAN
*K. pneumoniae*	2011			**62 (47–79)**	*8 (2–27)*		16 (6–32)	42 (37–50)	**61.5 (56–76)**		
	2012			**51 (18.1–71.7)**	*10 (6.9–17.7)*		16 (15–22.14)	34 (29–39.7)	**55 (52.3–63)**		
	2013			**52.5 (47–58)**	19.5 (18–21)		13.5 (12–15)	35.5 (34–37)	**70 (54–76)**		
	2014			**63 (51–75)**	14.5 (14–15)		18.5 (18–19)	31 (29–33)	**60.5 (54–67)**		
	2015			**52.5 (47–58)**	22 (22–22)		17 (17–17)	35 (35–35)	**51.5 (50–53)**		
	2016			**58 (53–63)**	19 (19–19)		18 (16–20)	33 (33–33)	**54 (50–58)**		
	2017			**67.5 (52–78)**	20 (14–32)		17.5 (15–20)	33 (33–53)	**60.5 (50–64)**		
	2018			**72 (54–78)**	17.5 (9–36)		19.5 (13–23)	48.5 (43–80)	**58 (51–90)**		
*E .coli*	2011		**88 (83.1–92.9)**	**59 (57–78)**	*1.5 (0.1–2.9)*	37 (28–46.7)	*4.5 (3–45)*	**66 (59–74)**	**70.5 (63.2–78.9)**		
	2012		**84 (79.3–91.5)**	**60 (55.4–75.3)**	*1 (0.5–2)*	38 (36–44.7)	*5 (3–14.7)*	**65 (36.8–73)**	**70 (65.2–75.8)**		
	2013		**85 (69–92)**	**63 (61–79)**	*5 (2–9)*	36 (33–40)	*5 (3–25)*	**67 (67–73)**	**75 (70–76)**		
	2014		**85 (81–88)**	**65 (64–74)**	*5 (2–15)*	37 (36–40)	*3 (3–24)*	**74 (68–75)**	**73 (68–82)**		
	2015		**89.5 (87–97)**	**68 (67–83)**	*5.5 (2–7)*	36.5 (36–43)	*4.5 (3–8)*	**69 (67–79)**	**70 (69–77)**		
	2016		**90 (89–91)**	**71.5 (66.5–78.3)**	*6.5 (5–9.8)*	42 (37–49)	*5 (4–7)*	**59 (10.9–72.5)**	**76 (71.3–80)**		
	2017		**95 (91–97)**	**76 (68–82)**	*10 (4–20)*	33 (24–34)	*10.5 (4–19)*	**65 (33–73)**	**66.5 (62–71)**		
	2018		**91 (89–93)**	**74 (64–84)**	*8.5 (6–15)*	32 (8–33)	*6 (4–25)*	**71.5 (67–80)**	**74 (67–90)**		
*Acinetobacter *spp.	2011				**87 (46.3–96.2)**	**59 (15.4–73.7)**	**74 (45.5–95.6)**				
	2012				**79 (52.1–89.1)**	**73 (49.3–82.4)**	**75 (48.1–82.1)**				
	2013				**85.5 (25–89)**	**73.5 (48–84)**	**73 (41–83)**				
	2014				**90 (64–95)**	**72 (40–95)**	**50 (24–88)**				
	2015				**70 (53–87)**	**51.5 (47–56)**	38.5 (36–41)				
	2016				**62 (51–73)**	46.5 (46–47)	36 (32–40)				
	2017				**70 (51–89)**	43 (35–66)	48.6 (37–55)				
	2018				**71 (54–88)**	**62 (53–71)**	**51 (48–54)**				
*Salmonella Typhi*	2011			*0*				*8 (3–13)*			
	2012			*0 (–)*				**73 (24–85)**			
	2013			*0 (–)*				**91 (70–92)**			
	2014			*0 (–)*				**88 (85–91)**			
	2015			*1 (0–2)*				**91 (91–91)**			
	2016			*0.1*				**89**			
	2017			29				**81**			
	2018			**58.5 (50–67)**				**99.5 (99–100)**			
*Shigella* spp.	2011			*6 (5–7)*				40.5 (22–59)			
	2012			18 (16–20)				17.5 (14–21)			
	2013			*9*				18			
	2014			13				16			
	2015			15				22			
	2016			18				15			
	2017			37				32			
	2018			35				23			
*N. gonnorheae*	2011			*0 (–)*				**92.5 (92–93)**			
	2012			*0 (–)*				**95 (93–97)**			
	2013			*0*				**93**			
	2014			*0*				**96**			
	2015			*0*				**95**			
	2016			*0*				**95**			
	2017			*0*				**86**			
	2018			NT				**96**			
*S. aureus*	2011									**50 (43–59)**	*0 (–)*
	2012									**55.5 (30.4–64.5)**	*0 (–)*
	2013									**55 (51–58)**	*0 (–)*
	2014									**56.5 (55–58)**	*0 (–)*
	2015									**59 (59–59)**	*0 (–)*
	2016									**60.5 (27–69)**	*0 (–)*
	2017									42 (29–67)	*0 (–)*
	2018									**65.5 (41–68)**	*0 (–)*
*S. pneumoniae*	2011	*4.5 (4–5)*							**65 (50–70)**		
	2012	*8 (3–20)*							**69 (67–100)**		
	2013	*4*							**77**		
	2014	*5*							**80**		
	2015	14.5 (0–29)							**75**		
	2016	16							**81**		
	2017	13							**71**		
	2018	16.5 (8–25)							**69 (59–79)**		

The pathogen/antimicrobial combination used was in accordance with WHO GLASS. Antibiograms published in Pakistan Antimicrobial Resistance Network (PARN) (2006–2018) were included. For *Staphylococcus aureus* in addition to the antimicrobials recommended for reporting in GLASS, vancomycin has also been included. Antimicrobial Resistance rates are shown as median of percentage resistance reported in published antibiograms. 95% confidence intervals are given in brackets. Resistance rates for 2017 were derived from the reported sensitivity rates, using the formulae = (Total no of isolates for which antimicrobial susceptibility testing was conducted − no of reported susceptible isolates/Total no of isolates for which antimicrobial susceptibility testing was conducted)*100%

Resistance was graded as: very low (0–10%): in *italic*; low (11–30%): plain font; moderate (30–50%): underlined; high (≥ 50%): **bold font**

*PEN* penicillin, *AMP* ampicillin, *CRO/CTM* ceftriaxone/cefotaxime, *CAZ* ceftazidime, *MEM/IPM* meropenem/imipenem, *GEN* gentamicin, *CIP* ciprofloxacin, *SXT* sulfamethoxazole and trimethoprim, *AK* amikacin, *OXA* oxacillin, *VAN* vancomycin

Additional files 2, 3, 4 and 5 complement Tables 2, 3, 4 and 5. These additional files provide resistance rates reported from each selected literature studies and published antibiogram.

### *Klebsiella pneumoniae* and *Escherichia coli*

Hospital-based studies consistently indicated high rates of resistance to 3rd generation cephalosporins, fluoroquinolones and cotrimoxazole amongst both *K. pneumoniae* and *E. coli* (Table 2). Meanwhile, moderate to high resistance to fluoroquinolones and co-trimoxazole and increasing resistance to 3rd generation cephalosporins is reported from laboratory surveillance, from community-based studies (2012–2018) (Table 3) and in the antibiogram data (Table 5). Information on resistant *K. pneumoniae* amongst the paediatric population is sparse. However, published data reporting resistance in this age group is available for *E. coli* suggesting moderate to high rates of resistance to 3rd generation cephalosporins, fluoroquinolones and high resistance rates to cotrimoxazole (Table 4). Carbapenem resistance rates of under 30% for both *K. pneumoniae* and *E. coli* were reported in published studies as well as in the antibiograms (Tables 2, 3, 4, and 5). With the antibiograms suggesting increasing resistance 2013–2018 (Table 5) and one ICU based study (Table 2) reporting 56% carbapenem resistance amongst their *K. pneumoniae* isolates [19].

### *Acinetobacter* species

High resistance rates to aminoglycosides (amikacin and gentamicin) and to carbapenems (Tables 2, 4, and 5) is documented for *Acinetobacter* species.

### *Salmonella* Typhi

Fluoroquinolone resistance rates of over 80% amongst *S.* Typhi in the country have been documented in published reports since 2014 (Table 3) and in antibiograms since 2012 (Table 5). The emergence of ceftriaxone resistant *S.* Typhi was captured in both publications as well as in antibiograms (Tables 3 and 5). Laboratory-based studies conducted between 2008 and 2013 documented very low rates of *S.* Typhi resistant to ceftriaxone [20–22]. In December 2016, an outbreak of ceftriaxone resistant *S*. Typhi or extensively drug resistant (XDR) *S.* Typhi was reported from the southern province of Sindh, as a result of CTX-M gene acquisition in the widely prevalent fluoroquinolone, ampicillin, cotrimoxazole and chloramphenicol resistant strain of *S*. Typhi. This outbreak was recorded in antibiogram data in 2017 (Table 5) and published a year later [23, 24].

### *Shigella* species

While strains showing resistance to ceftriaxone have been reported, the rate of such resistance remains low (Table 3). A laboratory-based study conducted in 2006–2007 reported an increase in resistance to ceftriaxone from 2 to 8%, and to ofloxacin from 4.3 to 10.9% over the study period [25]. This study further reported that ceftriaxone resistance was highest amongst *Shigella flexineri* followed by *Shigella sonnei* [25]. Similar figures were reported from another study conducted between 2011 and 2013 on isolates (n = 45) from cancer patients documenting resistance rates of 7% for ceftriaxone and 25% for ciprofloxacin [26]. Information from published studies is supported by the antibiogram data reporting low rate of resistance amongst *Shigella* species to both ceftriaxone and ciprofloxacin (2011–2016). Subsequent data however reports an increase in resistance rates to moderate levels for ceftriaxone (2017–2018) and for ciprofloxacin in 2017 (Table 5).

### *Neisseria gonorrhoeae*

Resistance amongst *N. gonorrhoeae* to cephalosporins, azithromycin or spectinomycin was not reported in either the published studies or in the antibiograms from 2006 to 2018. However high fluoroquinolone resistance rates were consistently reported (Tables 3 and 5).

### *Staphylococcus aureus*

Heat map for the years 2007–2014 (Table 2) shows an increase in prevalence of hospital acquired methicillin resistant *S. aureus* (MRSA) from low (< 30%) in 2008 [27] to moderate levels (> 50%) in 2017 [28]. MRSA rates in community and laboratory surveillance data (Table 3) also remained low to moderate; 12% in 2005–2008 [29] to 28% from 2009 to 2010 [29]. However, antibiogram data show high median MRSA rates (Table 5).

### *Streptococcus pneumoniae*

Published reports from the country for resistance amongst *S. pneumoniae* strains are scant. However, a study [30] based on data from 2013 to 2014 reported high rates of resistance to penicillin and cotrimoxazole, and moderate resistance to ceftriaxone (Table 3). These findings are in agreement with antibiograms showing increasing resistance to penicillin between 2015 and 2018, and high rate of cotrimoxazole resistance between 2011 and 2018 (Table 5).

### Antimicrobial resistance based on patients’ demographics

A few studies [18, 28, 31–33] disaggregated frequency of drug resistant isolates based on age and or gender. Jadoon et al. (2015) [31] reported higher frequency of ciprofloxacin resistant *E. coli* in males (61%) as compared to females (32.8%). Whereas, Ali et al. (2017) [18] reported fluoroquinolone resistant *E. coli* to be higher in female patients (75–83.3%), aged 1–15 years, compared to male patients (44.4%). In age groups ≥ 16 years similar frequency of fluoroquinolone resistant isolates was reported in both genders. Kalam et al. (2014) [32] reported higher frequency of MDR Gram negative bacteria in males (66%) compared to females (34.2%). Similarly, MRSA were found to be present at a higher frequency in adult males (70%) compared to female patients (30%) [28]. A study conducted in the Pediatric Intensive Care Unit (PICU) found that amongst male patients (aged 1 month–15 years) MDR Gram negative infection occurred most frequently in age group < 1 year (52.7%) [33]. Socioeconomic status of the patients was not reported in the reviewed studies.

Only two studies shared outcome data of patients with drug resistant infection. Kalam et al. (2014) [32] reported deaths in 46.2% of patients with Gram negative bacterial infection. Similarly, 42.9% of critically ill pediatric patients receiving intravenous polymyxin B with MDR Gram negative infection expired [33].

## Discussion

Our data provides a window into the gradual emergence and spread of antimicrobial resistance in Pakistan from 2009 to 2018. During this period both published reports as well as laboratory based antibiogram data reveal increasing resistance to antimicrobial agents amongst GLASS priority pathogens in the country. The number of studies reporting resistance rates from children were few, but those available were consistent with findings from adult populations. These findings are also consistent with recent publications reporting increasing AMR in the region [73–76].

Widespread resistance to fluoroquinolones together with rapid increase in Carbapenem resistant Enterobacteriaceae (CRE) over the past decade is widely reported from South Asia [73, 74, 76–79]. Colistin is frequently used as a last resort antibiotic in such cases. However, the recommended susceptibility testing method for colistin has recently been revised. Therefore, while high colistin resistance is reported in the literature [75], given the concern that the reported rates may not be based on the recommended methods, colistin resistance data was not included.

The quality and standardization of antimicrobial sensitivity testing methodologies used in laboratories across the country vary. This is likely to be reflected in all AMR related data including from published studies as well as from antibiogram based information. Additionally, antibiograms submitted to PARN website are on a voluntary basis, therefore the data presented is limited by the information available for that year. Frequency of antimicrobial resistance in accordance with gender and age groups was reported in a few studies [18, 28, 31–33]. However published literature [80–82] indicate that age, gender, comorbid conditions and underlying factors such as: previous use of antibiotics and duration of in-dwelling catheters have a significant role. That for patients with urinary tract infection caused by *E. coli*, amikacin, nitrofurantoin and colistin, age and gender should be considered while prescribing antibacterials [81]. Similarly, Bruie et al. (2004) [80], found female patients to be at an increased risk of penicillin resistant *S. pneumoniae* infection. Further, their study indicates that presence of HIV increases the risk of AMR in pneumococcal infections. Such information can provide an evidence-base for physicians to avoid unnecessary antibiotics for critical patients.

Hospital settings including intensive care units (ICU) reported relatively high resistance to fluoroquinolones amongst *K. pneumoniae* and *E. coli*, as well as resistance amongst these organisms to cephalosporins and carbapenems with an increase in resistance being reported for *K. pneumoniae*. These data support published reports [83–85] enforcing concern about role of hospitals in contributing to spread of resistance.

The few community-based reports available from the country, endorsed hospital and laboratory-based findings for high AMR rates. These findings are also supported by a systematic review and meta-analysis reporting moderate to high levels of antimicrobial resistance amongst bacterial pathogens associated with community acquired paediatric bloodstream infections in low- and middle-income countries [86] as well as by increasing reports of community acquired AMR globally including from LMIC [23, 87–89]. The paucity of community-based AMR reports in our study points not only to a weakness in the AMR data being presented, but also to a dependence on hospital and laboratory-based studies for information on AMR in LMICs and to the challenges of capturing community-based information on AMR in these settings.

Surveillance using GLASS as well as PASS relies on information from select surveillance sites which is then combined as national data. While this system is valuable at the macro level, the combined data overlooks the granularity and local information required by treating physicians. As such antibiograms are a useful means of sharing antimicrobial resistance information at a local level. Therefore, a compilation of antibiogram data may also provide useful information at a national level. As the country moves towards strengthening AMR surveillance, contribution of published antibiograms should be explored towards supplementing national surveillance efforts.

It was encouraging to note that hospital and laboratory antibiogram based information from the PARN website agreed with, and complemented published reports well suggesting that such information constitutes an important source of AMR data, which needs to be harnessed, and utilized in national AMR analyses. We observed rare discrepancies between published data and antibiograms viz in the case of *S. aureus*. Higher median resistance in *S. aureus* in antibiograms is likely driven by a predominance of hospital and ICU data.

More importantly such antibiogram data also correlated well with the AMR information from Pakistan included in the GLASS report 2017–2018 [90]. This is not unexpected as GLASS data reported from many LMICs is primarily reliant on laboratory-based data as well.

National AMR surveillance efforts which provide information to the GLASS platform currently focuses on collecting resistance data on select organisms from specified enrolled sites. Such efforts while essential, have limitations; they include only select laboratories connected to the surveillance system, information supplied is limited to specific pathogens included in GLASS. As such localized resistance patterns and geographic distribution are difficult to assess. We therefore propose that national level data, such as that collected for GLASS, be supplemented with individual hospital antibiograms based information to inform sub-national AMR rates. This concept is similar to that of ResistanceMap [91]; an open-access online resource reporting resistance data from 66 countries from 1999 to 2017, and also to Resistancebank [92]; an online repository created in 2019 for surveillance data on animal antimicrobial resistance.

Based on our findings we propose similar initiatives at a national level offering a central platform for sharing antibiograms, allowing comparison of susceptibility rates at sub-national levels to create opportunities for information sharing, monitoring of AMR, and focused control efforts.

## Conclusion

Data from both published studies and from antibiograms were complementary in showing high resistance to most antibacterials studied. These included third generation cephalosporins and carbapanems. Gaps in literature including paucity of information on AMR amongst peadiatric and community based populations, as well as a lack of studies exploring the association between patient demographic, underlying co-morbidities and resistance are highlighted by our study. We further show that antibiograms are a valuable tool to aid physicians in understanding resistance rates locally towards improving prescribing practices.

## Supplementary Information

**Additional file 1.** Terms used for searching peer reviewed literature from electronic database Medline (PubMed).

**Additional file 2.** Antimicrobial resistance rates (shown as percent resistance) reported in hospital-based studies (2006–2017).

**Additional file 3.** Antimicrobial resistance rates (shown as percent resistance) from laboratory surveillance and community-based literature review (2009–2018).

**Additional file 4.** Antimicrobial resistance rates (shown as percent resistance) amongst pediatric population based on literature review (2010–2017).

**Additional file 5.** Antimicrobial resistance rates from laboratory based antibiograms (2006–2018).

## Data Availability

All data generated or analysed during this study are provided in the manuscript and supplementary information files. PARN is openly available to the public. The papers can be accessed through the journal links on PubMed. Most of these articles are open access. Resistance rates can be also accessed through the referenced studies available on PubMed (https://pubmed.ncbi.nlm.nih.gov/). Antibiogram data can be accessed through the following URL: http://parn.org.pk/antimicrobial-data/.

## Abbreviations

AMR: Antimicrobial resistance
ARG: Antimicrobial resistance genes
CLSI: Clinical Laboratory Standards Institute
CRE: Carbapenem resistant Enterobacteriaceae
GDP: Gross Domestic Product
GLASS: Global Antimicrobial Surveillance System
PASS: Pakistan AMR surveillance system
NDM: New Delhi Metallo-betalactamase
XDR: Extensively drug resistant
MDR: Multi drug resistant
QC: Quality control
RCT: Randomized control trial
ICU: Intensive Care Unit
MICU: Medical Intensive Care Unit
MRSA: Methicillin Resistant *Staphylococcus aureus*
PICU: Pediatric Intensive Care Unit
LMIC: Low Middle Income Countries
