# Supplementary material for: Antimicrobial resistance among GLASS priority pathogens from Pakistan: 2006–2018
Source: BMC Infect Dis. 2021 Dec 7;21:1231. doi: 10.1186/s12879-021-06795-0 (PMC8650393; doi:10.1186/s12879-021-06795-0)
Supplement: Supplementary file 2 — Additional file 2. Antimicrobial resistance rates (shown as percent resistance) reported in hospital-based studies (2006–2017). [file 12879_2021_6795_MOESM2_ESM.docx]

**Additional File 2. Antimicrobial Resistance Rates (shown as percent resistance) reported in hospital-based studies (2006-2017).**

| **Priority**  **bacteria** | **Year of publication** | **Duration**  **of study** | **N** | **AMP** | **CRO/**  **CTM** | **CAZ** | **MEM/**  **IPM** | **GEN** | **AK** | **CIP/**  **OFX** | **SXT** | **TIG** | **MIN** | **OXA** | **VAN** | **Ref** |
| --- | --- | --- | --- | --- | --- | --- | --- | --- | --- | --- | --- | --- | --- | --- | --- | --- |
| ***K. pneumoniae*** | 2009 | 2006-7 | 9 |  | 100 |  | 0 |  |  |  | 100 |  |  |  |  | [34] |
|  | 2010 | 2007-8 | 72 |  | 62.5 | 47.2 | 20.8 |  |  | 73.6 | 88.9 |  |  |  |  | [35] |
|  | 2010 | 2002-7 | 15914 |  |  |  | 0.4 |  |  | 22.4 | 41.25 |  |  |  |  | [36] |
|  | 2016 | 2015-16 | 58 |  | 94 |  | 56 |  |  | 71 | 73 |  |  |  |  | [19] |
|  | 2017 | 2013-14 | 93 |  | 60 |  | 1.1 |  |  |  | 68.5 |  |  |  |  | [37] |
|  | Total(N) | | **368** |  |  |  |  |  |  |  |  |  |  |  |  |  |
| ***E. coli*** | 2009 | 2008 | 29 | 86.2 | 82.4 |  |  |  |  | 75.9 |  |  |  |  |  | [38] |
|  | 2010 | 2007- 8 | 53 |  | 71.7 | 71.7 | 15.1 |  |  | 75.5 | 64.2 |  |  |  |  | [35] |
|  | 2010 | 2009 | 46 |  | 78.3 | 55.5 | 5.1 |  |  | 65.2 | 78.3 |  |  |  |  | [39] |
|  | 2015 | 2011-12 | 166 |  |  |  |  |  |  | 59.1 |  |  |  |  |  | [31] |
|  | 2015 | 2012-13 | 227 | 96.5 | 80.2 |  | 7 |  |  | 78.4 |  |  |  |  |  | [40] |
|  | 2016 | 2013-14 | 89 |  |  |  | 5.6 |  |  | 57.3 | 61.8 |  |  |  |  | [41] |
|  | 2016 | 2014 | 50 | 98 | 58 |  | 12 |  |  | 60 | 60 |  |  |  |  | [42] |
|  | 2016 | 2015-16 | 87 | 93 | 93 |  | 10 |  |  | 84 |  |  |  |  |  | [19] |
|  | 2017 | 2013-14 | 108 |  |  |  | 1.89 |  |  | 74.04 | 76 |  |  |  |  | [37] |
|  | 2017 | 2014-15 | 351 | 90.8 | 78 | 45.8 | 2.2 |  |  | 79.3 | 67.2 |  |  |  |  | [43] |
|  | 2018 | 2017 | 119 | 71.4 | 65.5 | 64.7 | 5 |  |  | 71.4 | 63.9 |  |  |  |  | [18] |
|  | Total (N) | | **1403** |  |  |  |  |  |  |  |  |  |  |  |  |  |
| ***Acinetobacter sp.*** | 2009 | 2009 | 27 |  |  |  | 85 | 92 | 78 |  |  |  |  |  |  | [34] |
|  | 2009 | 2006 | 4 |  |  |  | 75 |  |  |  |  |  |  |  |  | [44] |
|  | 2014 | 2010-11 | 90 |  |  |  | 65.5 |  | 68.94 |  |  | 20 | 83.3 |  |  | [45] |
|  | 2014 | 2010 | 30 |  |  |  | 53.3 |  |  |  |  |  |  |  |  | [32] |
|  | 2015 | 2011 | 26 |  |  |  | 96.2 |  | 50 |  |  | 11.5 |  |  |  | [46] |
|  | 2016 | 2014 | 51 |  |  |  | 17.6 |  | 37.2 |  |  |  |  |  |  | [42] |
|  | 2016 | 2013-14 | 8 |  |  |  | 100 | 100 | 75 |  |  |  |  |  |  | [41] |
|  | 2016 | 2015-16 | 87 |  |  |  | 100 | 97 | 95 |  |  |  |  |  |  | [19] |
|  | Total (N) | | **354** |  |  |  |  |  |  |  |  |  |  |  |  |  |
| ***S. aureus*** | 2009 | 2005-8 | 195 |  |  |  |  |  |  |  |  |  |  | 21.5 | 0 | [27] |
|  | 2014 | 2009-10 | 54 |  |  |  |  |  |  |  |  |  |  | 48.1 |  | [29] |
|  | 2014 | 2011 | 375 |  |  |  |  |  |  |  |  |  |  | 33.9 |  | [47] |
|  | 2014 | 2011 | 177 |  |  |  |  |  |  |  |  |  |  | 63.27 |  | [48] |
|  | 2015 | -- | 104 |  |  |  |  |  |  |  |  |  |  | 49.04 |  | [49] |
|  | 2015 | 2013-14 | 77 |  |  |  |  |  |  |  |  |  |  | 2.9 |  | [50] |
|  | 2017 | 2015-17 | 346 |  |  |  |  |  |  |  |  |  |  | 52 |  | [28] |
|  | 2017 | 2013-14 | 142 |  |  |  |  |  |  |  |  |  |  | 26.6 |  | [37] |
|  | 2017 | 2013 | 26 |  |  |  |  |  |  |  |  |  |  | 84.6 |  | [51] |
|  | Total (N) | | **2458** |  |  |  |  |  |  |  |  |  |  |  |  |  |

**Legend:** The pathogen/ antimicrobial combination used was in accordance with WHO GLASS.

n value: Number of isolates included in the study reported

**Abbreviations:** AMP= Ampicillin, CRO/CTM= Ceftriaxone/ Cefotaxime, CAZ= Ceftazidime, MEM/IPM= Meropenem/Imipenem, GEN= Gentamicin, AK= Amikacin, CIP/OFX= Ciprofloxacin/ Ofloxacin, SXT= Sulfamethoxazole and trimethoprim, TIG= Tigecycline, MIN= Minocycline, OXA= Oxacillin VAN= Vancomycin. sp.=species.

For Staphylococcus aureus in addition to the antimicrobials recommended for reporting in GLASS, vancomycin has also been included.
