# Supplementary material for: Antimicrobial resistance among GLASS priority pathogens from Pakistan: 2006–2018
Source: BMC Infect Dis. 2021 Dec 7;21:1231. doi: 10.1186/s12879-021-06795-0 (PMC8650393; doi:10.1186/s12879-021-06795-0)
Supplement: Supplementary file 3 — Additional file 3. Antimicrobial resistance rates (shown as percent resistance) from laboratory surveillance and community-based literature review (2009–2018). [file 12879_2021_6795_MOESM3_ESM.docx]

| **Priority**  **bacteria** | **Year of publication** | **Duration**  **of study** | **n** | **PEN** | **AMP** | **CRO/**  **CTM** | **CAZ** | **MEM/**  **IPM** | **AK** | **CIP/OFX** | **SXT** | **AZM** | **SPT** | **OXA** | **VAN** | **Ref** |
| --- | --- | --- | --- | --- | --- | --- | --- | --- | --- | --- | --- | --- | --- | --- | --- | --- |
| ***K. pneumoniae*** | 2012 | 2010-11 | 672 |  |  |  |  |  |  | 34.5 |  |  |  |  |  | [52] |
|  | 2013 | 2008-11 | 1617 |  |  | 26.6 | 26.7 | 1.4 |  | 53.8 |  |  |  |  |  | [53] |
|  | 2016 | 2012-13 | 60 |  |  | 21.7 |  |  |  |  |  |  |  |  |  | [20] |
|  | 2018 | 2015 | 59 |  |  | 39 |  | 15 | 10 | 47.5 | 51 |  |  |  |  | [54] |
|  | **Total(N)** | | **2426** |  |  |  |  |  |  |  |  |  |  |  |  |  |
| ***E. coli*** | 2012 | 2010-11 | 1290 |  |  |  |  |  |  | 58.4 |  |  |  |  |  | [52] |
|  | 2015 | -- | 20 |  | 90 |  |  |  |  | 50 | 55 |  |  |  |  | [55] |
|  | 2016 | 2012-13 | 110 |  |  | 10 |  |  |  |  |  |  |  |  |  | [20] |
|  | 2018 | 2015 | 188 |  | 97 | 58.5 |  | 6 |  | 65 | 70 |  |  |  |  | [54] |
|  | **Total (N)** | | **1608** |  |  |  |  |  |  |  |  |  |  |  |  |  |
| ***S.* Typhi** | 2012 | 2008-10 | 131 |  |  | 1.3 |  |  |  |  |  |  |  |  |  | [21] |
|  | 2014 | 2009-11 | 2576 |  |  | 0.08 |  |  |  | 88.2 |  |  |  |  |  | [22] |
|  | 2016 | 2012-13 | 40 |  |  | 7.5 |  |  |  |  |  |  |  |  |  | [20] |
|  | 2017 | 2014-15 | 270 |  |  | 0 |  |  |  | 93 |  |  |  |  |  | [56] |
|  | 2018 | 2012-14 | 1979 |  |  | 0.2 |  |  |  | 90 |  |  |  |  |  | [57] |
|  | **Total(N)** | | **390** |  |  |  |  |  |  |  |  |  |  |  |  |  |
| ***Shigella*** | 2009 | 2006 | 115 |  |  | 2 |  |  |  | 4.3 | 73.9 |  |  |  |  | [25] |
|  | 2009 | 2007 | 101 |  |  | 8 |  |  |  | 10.9 | 83.2 |  |  |  |  | [25] |
|  | 2016 | 2011-13 | 45 |  |  | 7 |  |  |  | 25 | 79 |  |  |  |  | [26] |
|  | **Total (N)** |  | **261** |  |  |  |  |  |  |  |  |  |  |  |  |  |
| ***N.gonorrhoeae*** | 2011 | 2007-9 | 112 |  |  |  |  |  |  | 98 |  |  |  |  |  | [58] |
|  | 2011 | 2007-9 | 318 |  |  |  |  |  |  | 89.6 |  |  |  |  |  | [59] |
|  | 2013 | 2008-11 | 18 |  |  | 0 |  |  |  | 93.8 |  | 7.7 | 0 |  |  | [60] |
|  | 2016 | 2012-14 | 100 |  |  | 0 |  |  |  | 86 |  | 1 | 0 |  |  | [61] |
|  | **Total (N)** | | **526** |  |  |  |  |  |  |  |  |  |  |  |  |  |
| ***S. aureus*** | 2009 | 2005-8 | 42 |  |  |  |  |  |  |  |  |  |  | 11.9 |  | [27] |
|  | 2014 | 2009-10 | 144 |  |  |  |  |  |  |  |  |  |  | 27.8 |  | [29] |
|  | **Total (N)** | | **815** |  |  |  |  |  |  |  |  |  |  |  |  |  |
| ***S. pneumoniae*** | 2016 | 2013-14 | 42 | 88.2 |  | 35.7 |  |  |  | 76.2 |  |  |  |  |  | [30] |

**Additional File 3 Antimicrobial Resistance Rates (shown as percent resistance) from laboratory surveillance and community-based literature review (2009-2018).**

**Legend:** The pathogen/ antimicrobial combination used was in accordance with WHO GLASS.

n value: Number of isolates included in the study reported

**Abbreviations:** PEN=Penicillin, AMP= Ampicillin, CRO/CTM= Ceftriaxone/ Ceftaxime, CAZ= Ceftazidime, MEM/IPM= Meropenem/Imipenem, GEN= Gentamicin, AMK= Amikacin, CIP= Ciprofloxacin, SXT= Sulfamethoxazole and trimethoprim, CT= Colistin, AZM= Azithromycin, SPT= Spectinomycin, OXA= Oxacillin VAN= Vancomycin., sp= species. For *Staphylococcus aureus* in addition to the antimicrobials recommended for reporting in GLASS, vancomycin has also been included.
