# Supplementary material for: Antimicrobial resistance among GLASS priority pathogens from Pakistan: 2006–2018
Source: BMC Infect Dis. 2021 Dec 7;21:1231. doi: 10.1186/s12879-021-06795-0 (PMC8650393; doi:10.1186/s12879-021-06795-0)
Supplement: Supplementary file 4 — Additional file 4. Antimicrobial resistance rates (shown as percent resistance) amongst pediatric population based on literature review (2010–2017). [file 12879_2021_6795_MOESM4_ESM.docx]

**Additional File 4. Antimicrobial Resistance Rates (shown as percent resistance) amongst pediatric population based on literature review (2010-2017).**

| **Priority Pathogens** | **Year of Study** | **Year of Publication** | **Category** | **n** | **AMP** | **CRO/**  **CTM** | **CAZ** | **MEM/**  **IPM** | **GEN** | **AK** | **CIP/**  **OFX** | **SXT** | **CLOX** | **VAN** | **Ref** |  |  |
| --- | --- | --- | --- | --- | --- | --- | --- | --- | --- | --- | --- | --- | --- | --- | --- | --- | --- |
| ***K. pneumoniae*** | 2006-11 | 2013 | I | 104 |  |  |  | 20 |  |  | 20 |  |  |  | [62] |  |  |
|  | Total(N) | | | 104 |  |  |  |  |  |  |  |  |  |  |  |  |  |
| ***E. coli*** | 2009-10 | 2010 | I | 30 | 73.3 | 63.4 | 50.3 | 2.9 |  |  | 40.3 |  |  |  | [63] |  |  |
|  | 2009-10 | 2012 | I | 30 | 73.3 | 46 | 46.2 | 20 |  |  | 40.3 |  |  |  | [64] |  |  |
|  | 2012-15 | 2016 | I | 811 | 89.8 | 78 |  | 5.3 |  |  | 32.7 |  |  |  | [65] |  |  |
|  | 2010-11 | 2016 | I | 35 | 80 |  |  |  |  |  | 68.57 | 77.1 |  |  | [66] |  |  |
|  | 2010-12 | 2016 | II | 46 | 91 |  |  |  | 40 |  | 39 | 78 |  |  | [67] |  |  |
|  | Total(N) | | | 952 |  |  |  |  |  |  |  |  |  |  |  |  | [64] |
| ***Acinetobacter* sp*.*** | 2009-10 | 2012 | I | 17 |  |  |  | 52.9 | 66.6 | 46.6 |  |  |  |  | [64] |  |  |
|  | 2010-11 | 2014 | I | 12 |  |  |  | 100 | 100 | 100 |  |  |  |  | [33] |  |  |
|  | 2014 | 2016 | I | 112 |  |  |  | 66 | 71.2 | 83.3 |  |  |  |  | [68] |  |  |
|  | 2014 | 2017 | I | 100 |  |  |  | 94.7 | 100 | 95.78 |  |  |  |  | [69] |  |  |
|  | Total(N) | | | 241 |  |  |  |  |  |  |  |  |  |  |  |  |  |
| ***S.* Typhi** | 2002-4 | 2012 | II | 189 |  | 0 |  |  |  |  | 0 |  |  |  | [70] |  |  |
|  | 2007-8 | 2010 | II | 16 |  | 0 |  |  |  |  | 46 |  |  |  | [71] |  |  |
|  | Total(N) |  |  | 205 |  |  |  |  |  |  |  |  |  |  |  |  |  |
| ***S. aureus*** | 2004-7 | 2011 | II | 304 |  |  |  |  |  |  |  |  | 4.3 |  | [72] |  |  |
|  | 2009-10 | 2012 | I | 35 |  |  |  |  |  |  |  |  |  | 0 | [64] |  |  |
|  | Total(N) | | | 339 |  |  |  |  |  |  |  |  |  |  |  |  |  |

**Legend:** The pathogen/ antimicrobial combination used was in accordance with WHO GLASS.

n value: Number of isolates included in the study reported

**Categories:** I: Hospital based studies, II: Lab-bases surveillance/Community studies

**Abbreviations used:** AMP= Ampicillin, CRO/CTM= Ceftriaxone/ Ceftaxime, CAZ= Ceftazidime, MEM/IPM= Meropenem/Imipenem, GEN= Gentamicin, AK= Amikacin, CIP/OFX= Ciprofloxacin/Ofloxacin, SXT= Sulfamethoxazole and trimethoprim, CLOX= Cloxacillin VAN= Vancomycin, sp.= species.
