# Supplementary material for: Antimicrobial resistance among GLASS priority pathogens from Pakistan: 2006–2018
Source: BMC Infect Dis. 2021 Dec 7;21:1231. doi: 10.1186/s12879-021-06795-0 (PMC8650393; doi:10.1186/s12879-021-06795-0)
Supplement: Supplementary file 5 — Additional file 5. Antimicrobial resistance rates from laboratory based antibiograms (2006–2018). [file 12879_2021_6795_MOESM5_ESM.docx]

**Additional File 5. Antimicrobial Resistance Rates from laboratory based antibiograms (2006-2018).**

| **Priority bacteria** | **Year** | **PEN** | **AMP** | **CRO/CTM** | **MEM/IPM** | **GEN** | **AK** | **CIP** | **SXT** | **OXA** | **VAN** |
| --- | --- | --- | --- | --- | --- | --- | --- | --- | --- | --- | --- |
| ***K. pneumoniae*** | **2011** |  |  | NT | 2 |  | 6 | NT | NT |  |  |
|  | **2011** |  |  | 79 | 13 |  | 20 | 37 | 76 |  |  |
|  | **2011** |  |  | 47 | 8 |  | 32 | 50 | 67 |  |  |
|  | **2011** |  |  | 72 | 27 |  | 16 | 42 | 56 |  |  |
|  | **2011** |  |  | 52 | 8 |  | 16 | NT | 56 |  |  |
|  | **2012** |  |  | 49 | 10 |  | 15 | 37 | 51 |  |  |
|  | **2012** |  |  | 51 | 8 |  | 15 | 34 | 55 |  |  |
|  | **2012** |  |  | 73 | 19 |  | 24 | 36 | 62 |  |  |
|  | **2012** |  |  | 4 | 6.5 |  | 16 | 41 | 55 |  |  |
|  | **2012** |  |  | 51 | 8 |  | 15 | 34 | 55 |  |  |
|  | **2012** |  |  | 69 | 15 |  | 18 | 29 | 63 |  |  |
|  | **2012** |  |  | 69 | 15 |  | 18 | 29 | 63 |  |  |
|  | **2013** |  |  | 51 | 14 |  | 18 | 34 | 54 |  |  |
|  | **2013** |  |  | 75 | 15 |  | 19 | 37 | 67 |  |  |
|  | **2014** |  |  | 58 | 18 |  | 15 | NT | NT |  |  |
|  | **2014** |  |  | 47 | 21 |  | 12 | NT | NT |  |  |
|  | **2015** |  |  | 52 | 22 |  | 17 | 35 | 50 |  |  |
|  | **2015** |  |  | 50 | 22 |  | 17 | 35 | 53 |  |  |
|  | **2016** |  |  | 53 | 19 |  | 20 | 33 | 50 |  |  |
|  | **2016** |  |  | 63 | 19 |  | 16 | 33 | 58 |  |  |
|  | **2017** |  |  | 71 | 32 |  | 16 | 33 | 58 |  |  |
|  | **2017** |  |  | 64 | 20 |  | 15 | 33 | 64 |  |  |
|  | **2017** |  |  | 78 | 20 |  | 19 | 53 | 63 |  |  |
|  | **2017** |  |  | 52 | 14 |  | 20 | 33 | 50 |  |  |
|  | **2018** |  |  | 72 | 9 |  | 19.5 | 48.5 | 51 |  |  |
|  | **2018** |  |  | 54 | 17.5 |  | 23 | 43 | 58 |  |  |
|  | **2018** |  |  | 78 | 36 |  | 23 | 80 | 90 |  |  |
| ***E.coli*** | **2011** |  | 92 | 78 | 3 | 47 | 4 | 74 |  |  |  |
|  | **2011** |  | 84 | 59 | 1 | 37 | 3 | 65 |  |  |  |
|  | **2011** |  | 85 | 77 | 2 | 37 | 5 | 59 |  |  |  |
|  | **2011** |  | 83 | 59 | 2 | 27 | 45 | 71 |  |  |  |
|  | **2011** |  | 93 | 57 | 1 | 44 | NT | 66 |  |  |  |
|  | **2011** |  | 91 | NT | 0 | 37 | NT | NT |  |  |  |
|  | **2012** |  | 83 | 50 | 9 | 79 | 0 | 61 |  |  |  |
|  | **2012** |  | 92 | 73 | 2 | 41 | 3 | 73 |  |  |  |
|  | **2012** |  | 93 | 80 | 2 | 45 | 6 | 73 |  |  |  |
|  | **2012** |  | 84 | 59 | 1 | 36 | 3 | 66 |  |  |  |
|  | **2012** |  | 84 | 59 | 0.75 | 36 | 3 | 65 |  |  |  |
|  | **2012** |  | 84 | 58 | 1 | 38 | 5 | 58 |  |  |  |
|  | **2012** |  | 79 | 61 | 1 | 40 | 21 | 21 |  |  |  |
|  | **2012** |  | 85 | 65 | 0.5 | 37 | 12 | 35 |  |  |  |
|  | **2012** |  | 70 | NT | 0 | 29 | 15 | 83 |  |  |  |
|  | **2013** |  | 92 | 79 | 5 | 40 | 3 | 73 | 76 |  |  |
|  | **2013** |  | 85 | 63 | 2 | 36 | 5 | 67 | 70 |  |  |
|  | **2013** |  | 91 | 75 | 1 | 35 | 5 | 62 | 75 |  |  |
|  | **2013** |  | 97 | 84 | 3 | 45 | 6 | 72 |  |  |  |
|  | **2013** |  | 69 | 61 | 9 | 33 | 25 | 67 |  |  |  |
|  | **2014** |  | 81 | 65 | 15 | 36 | 24 | 75 | 82 |  |  |
|  | **2014** |  | 88 | 74 | 5 | 40 | 3 | 74 | 73 |  |  |
|  | **2014** |  | 85 | 64 | 2 | 37 | 3 | 68 | 68 |  |  |
|  | **2015** |  | 88 | 68 |  | 36 | 3 | 68 | 70 |  |  |
|  | **2015** |  | 87 | 67 | 5 | 36 | 3 | 67 | 70 |  |  |
|  | **2016** |  | 91 | 79 | 10 | 37 | 6 | 73 | 74 |  |  |
|  | **2016** |  | 89 | 66 | 5 | 37 | 4 | 68 | 71 |  |  |
|  | **2016** |  | NT | 72 | 5 | 49 | 4 | 55 | 80 |  |  |
|  | **2016** |  | NT | 71 | 8 | 42 | 7 | 63 | 76 |  |  |
|  | **2017** |  | 95 | 82 | 14 | 34 | 6 | 65 | 71 |  |  |
|  | **2017** |  | 91 | 68 | 4 | 33 | 4 | 73 | 69 |  |  |
|  | **2017** |  | NT | 76 | 6 | 24 | 19 | 65 | 62 |  |  |
|  | **2018** |  | 91 | 84 | 8 | 8 | 6 | 80 | 90 |  |  |
|  | **2018** |  | 93 | 74 | 15 | 33 | 4 | 71.5 | 74 |  |  |
|  | **2018** |  | 89 | 64 | 6 | 32 | 25 | 67 | 67 |  |  |
| ***Acinetobacter sp.*** | **2011** |  |  |  | 87 | 73 | 43 |  |  |  |  |
|  | **2011** |  |  |  | 46 | 73 | 59 |  |  |  |  |
|  | **2011** |  |  |  | 100 | 59 | 100 |  |  |  |  |
|  | **2011** |  |  |  | 87 | 55 | 51 |  |  |  |  |
|  | **2011** |  |  |  | 47 | 0 | 74 |  |  |  |  |
|  | **2012** |  |  |  | 90 | 79 | 80 |  |  |  |  |
|  | **2012** |  |  |  | 79 | 75 | 74 |  |  |  |  |
|  | **2012** |  |  |  | 59 | 58 | 57 |  |  |  |  |
|  | **2012** |  |  |  | 49 | 49 | 44 |  |  |  |  |
|  | **2012** |  |  |  | 79 | 73 | 77 |  |  |  |  |
|  | **2012** |  |  |  | 75 | 50 | 83 |  |  |  |  |
|  | **2012** |  |  |  | 87 | 84 | 75 |  |  |  |  |
|  | **2013** |  |  |  | 87 | 82 | 82 |  |  |  |  |
|  | **2013** |  |  |  | 84 | 65 | 83 |  |  |  |  |
|  | **2013** |  |  |  | 89 | 48 | 64 |  |  |  |  |
|  | **2013** |  |  |  | 25 | 84 | 41 |  |  |  |  |
|  | **2014** |  |  |  | 90 | 72 | 50 |  |  |  |  |
|  | **2014** |  |  |  | 64 | 40 | 24 |  |  |  |  |
|  | **2014** |  |  |  | 95 | 95 | 88 |  |  |  |  |
|  | **2015** |  |  |  | 87 | 56 | 41 |  |  |  |  |
|  | **2015** |  |  |  | 53 | 47 | 36 |  |  |  |  |
|  | **2016** |  |  |  | 73 | 46 | 40 |  |  |  |  |
|  | **2016** |  |  |  | 51 | 47 | 32 |  |  |  |  |
|  | **2017** |  |  |  | 70 | 35 | 55 |  |  |  |  |
|  | **2017** |  |  |  | 89 | 66 | 54 |  |  |  |  |
|  | **2017** |  |  |  | 51 | 43 | 37 |  |  |  |  |
|  | **2018** |  |  |  | 88 | 71 | 54 |  |  |  |  |
|  | **2018** |  |  |  | 54 | 53 | 48 |  |  |  |  |
| ***Salmonella* Typhi** | **2011** |  |  | 0 |  |  |  | 13 |  |  |  |
|  | **2011** |  |  | 0 |  |  |  | 3 |  |  |  |
|  | **2012** |  |  | 0.7 |  |  |  | 73 |  |  |  |
|  | **2012** |  |  | 0 |  |  |  | 75 |  |  |  |
|  | **2012** |  |  | 0 |  |  |  | 85 |  |  |  |
|  | **2012** |  |  | 0 |  |  |  | 24 |  |  |  |
|  | **2012** |  |  | 0 |  |  |  | NT |  |  |  |
|  | **2012** |  |  | 0 |  |  |  | 29 |  |  |  |
|  | **2013** |  |  | 70 |  |  |  | 0 |  |  |  |
|  | **2013** |  |  | 92 |  |  |  | 0 |  |  |  |
|  | **2013** |  |  | 91 |  |  |  | 0 |  |  |  |
|  | **2014** |  |  | 91 |  |  |  | 0 |  |  |  |
|  | **2014** |  |  | 85 |  |  |  | 0 |  |  |  |
|  | **2015** |  |  | 0 |  |  |  | 91 |  |  |  |
|  | **2015** |  |  | 2 |  |  |  | 91 |  |  |  |
|  | **2016** |  |  | 0.1 |  |  |  | 89 |  |  |  |
|  | **2017** |  |  | 29 |  |  |  | 81 |  |  |  |
|  | **2018** |  |  | 50 |  |  |  | 99 |  |  |  |
|  | **2018** |  |  | 67 |  |  |  | 100 |  |  |  |
| ***Shigella*** | **2011** |  |  | 5 |  |  |  | 59 |  |  |  |
|  | **2011** |  |  | 7 |  |  |  | 22 |  |  |  |
|  | **2012** |  |  | 20 |  |  |  | 14 |  |  |  |
|  | **2012** |  |  | 16 |  |  |  | 21 |  |  |  |
|  | **2013** |  |  | 9 |  |  |  | 18 |  |  |  |
|  | **2014** |  |  | 13 |  |  |  | 16 |  |  |  |
|  | **2015** |  |  | 15 |  |  |  | 22 |  |  |  |
|  | **2016** |  |  | 18 |  |  |  | 15 |  |  |  |
|  | **2017** |  |  | 37 |  |  |  | 32 |  |  |  |
|  | **2018** |  |  | 35 |  |  |  | 23 |  |  |  |
| ***N. gonnorheae*** | **2011** |  |  | 0 |  |  |  | 92 |  |  |  |
|  | **2011** |  |  | 0 |  |  |  | 93 |  |  |  |
|  | **2012** |  |  | 0 |  |  |  | 93 |  |  |  |
|  | **2012** |  |  | 0 |  |  |  | 97 |  |  |  |
|  | **2013** |  |  | 0 |  |  |  | 100 |  |  |  |
|  | **2014** |  |  | 0 |  |  |  | 96 |  |  |  |
|  | **2015** |  |  | 0 |  |  |  | 95 |  |  |  |
|  | **2016** |  |  | 0 |  |  |  | 95 |  |  |  |
|  | **2017** |  |  | 0 |  |  |  | 86 |  |  |  |
|  | **2018** |  |  | NT |  |  |  | 96 |  |  |  |
| **S. aureus** | **2011** |  |  |  |  |  |  |  |  | 59 | 0 |
|  | **2011** |  |  |  |  |  |  |  |  | 52 | 0 |
|  | **2011** |  |  |  |  |  |  |  |  | 43 | 0 |
|  | **2011** |  |  |  |  |  |  |  |  | 43 | 0 |
|  | **2011** |  |  |  |  |  |  |  |  | 50 | 0 |
|  | **2011** |  |  |  |  |  |  |  |  | 0 | 0 |
|  | **2012** |  |  |  |  |  |  |  |  | 45 | 0 |
|  | **2012** |  |  |  |  |  |  |  |  | 58 | 0 |
|  | **2012** |  |  |  |  |  |  |  |  | 55 | 0 |
|  | **2012** |  |  |  |  |  |  |  |  | 47 | 0 |
|  | **2012** |  |  |  |  |  |  |  |  | 56 | 0 |
|  | **2012** |  |  |  |  |  |  |  |  | 59 | 0 |
|  | **2012** |  |  |  |  |  |  |  |  | 76 | 0 |
|  | **2012** |  |  |  |  |  |  |  |  | 58 | 0 |
|  | **2013** |  |  |  |  |  |  |  |  | 51 | 0 |
|  | **2013** |  |  |  |  |  |  |  |  | 55 | 0 |
|  | **2013** |  |  |  |  |  |  |  |  | 58 | 0 |
|  | **2014** |  |  |  |  |  |  |  |  | 55 | 0 |
|  | **2014** |  |  |  |  |  |  |  |  | 59 | 0 |
|  | **2015** |  |  |  |  |  |  |  |  | 59 | 0 |
|  | **2015** |  |  |  |  |  |  |  |  | 27 | 0 |
|  | **2016** |  |  |  |  |  |  |  |  | 61 | 0 |
|  | **2016** |  |  |  |  |  |  |  |  | 69 | 0 |
|  | **2016** |  |  |  |  |  |  |  |  | 60 | 0 |
|  | **2016** |  |  |  |  |  |  |  |  | 49 | 0 |
|  | **2017** |  |  |  |  |  |  |  |  | 67 | 0 |
|  | **2017** |  |  |  |  |  |  |  |  | 35 | 0 |
|  | **2017** |  |  |  |  |  |  |  |  | 41 | 0 |
|  | **2018** |  |  |  |  |  |  |  |  | 41 | 0 |
|  | **2018** |  |  |  |  |  |  |  |  | 68 | 0 |
| ***S. pneumoniae*** | **2011** | 4 |  |  |  |  |  |  | 50 |  |  |
|  | **2011** | 5 |  |  |  |  |  |  | 70 |  |  |
|  | **2012** | NT |  |  |  |  |  |  | 92 |  |  |
|  | **2012** | 5 |  |  |  |  |  |  | 69 |  |  |
|  | **2012** | 3 |  |  |  |  |  |  | 69 |  |  |
|  | **2012** | 11 |  |  |  |  |  |  | 67 |  |  |
|  | **2012** | 20 |  |  |  |  |  |  | 100 |  |  |
|  | **2013** | 4 |  |  |  |  |  |  | 77 |  |  |
|  | **2014** | 5 |  |  |  |  |  |  | 80 |  |  |
|  | **2015** | 29 |  |  |  |  |  |  | NT |  |  |
|  | **2015** | 0 |  |  |  |  |  |  | 75 |  |  |
|  | **2016** | 16 |  |  |  |  |  |  | 81 |  |  |
|  | **2017** | 13 |  |  |  |  |  |  | 71 |  |  |
|  | **2018** | 8 |  |  |  |  |  |  | 59 |  |  |
|  | **2018** | 25 |  |  |  |  |  |  | 79 |  |  |

**Legend:** Antimicrobial Resistance Rates expressed as percent resistance amongst the isolates reported in the antibiograms

**Abbreviations:** PEN= Penicillin, AMP= Ampicillin, CRO/CTM= Ceftriaxone/ Ceftaxime, MEM/IPM= Meropenem/Imipenem, GEN= Gentamicin, AK= Amikacin, CIP= Ciprofloxacin, SXT= Sulfamethoxazole and trimethoprim, OXA= Oxacillin VAN= Vancomycin. For *Staphylococcus aureus* in addition to the antimicrobials recommended for reporting in GLASS, vancomycin has also been included. N.T= not tested
