# Supplementary material for: Antimicrobial resistance among GLASS priority pathogens from Pakistan: 2006–2018
Source: BMC Infect Dis. 2021 Dec 7;21:1231. doi: 10.1186/s12879-021-06795-0 (PMC8650393; doi:10.1186/s12879-021-06795-0)
Supplement: Supplementary file 1 — Additional file 1. Terms used for searching peer reviewed literature from electronic database Medline (PubMed). [file 12879_2021_6795_MOESM1_ESM.docx]

**Additional File 1 Terms used for searching peer reviewed literature from electronic database Medline (PubMed)**

| **Selected bacteria** | **Search terms** |
| --- | --- |
| *K. pneumoniae* | **("klebsiella pneumoniae"[MeSH Terms] OR ("klebsiella"[All Fields] AND "pneumoniae"[All Fields]) OR "klebsiella pneumoniae"[All Fields]) AND (("carbapenems"[MeSH Terms] OR "carbapenems"[All Fields] OR "carbapenem"[All Fields]) AND Resistance [All Fields]) AND ("pakistan"[MeSH Terms] OR "pakistan"[All Fields]) AND (("2006/01/01"[PDat]: "2018/1/31"[PDat]))**  MeSH Terms: carbapenems; pakistan; klebsiella pneumoniae  **antimicrobial resistance in Klebsiella pneumoniae in Pakistan**  papx: anti infective agents  MeSH Terms: klebsiella pneumoniae; pakistan; anti infective agents |
| *E.coli* | antimicrobial resistance in E.coli from Pakistan AND ( "2006/01/01"[PDat] : "2018/01/31"[PDat] ) AND Humans[Mesh]  MeSH Terms: humans; pakistan; anti infective agents; escherichia coli  papx: anti infective agents |
| *Acinetobacter species* | **carbapenem resistance in Acinetobacter species from Pakistan**  MeSH Terms: pakistan; carbapenems; Acinetobacter  **antimicrobial resistance in Acinetobacter species in Pakistan** |
| Typhoidal *Salmonella* | **(("anti-infective agents"[Pharmacological Action] OR "anti-infective agents"[MeSH Terms] OR ("anti-infective"[All Fields] AND "agents"[All Fields]) OR "anti-infective agents"[All Fields] OR "antimicrobial"[All Fields]) AND resistance[All Fields] AND ("salmonella typhi"[MeSH Terms] OR ("salmonella"[All Fields] AND "typhi"[All Fields]) OR "salmonella typhi"[All Fields]) AND ("pakistan"[MeSH Terms] OR "pakistan"[All Fields])) AND ("2006/01/01"[PDAT] : "2018/1/31"[PDAT])**  MeSH Terms: anti infective agents; pakistan; salmonella typhi  papx: anti infective agents |
| *Shigella* species | **antimicrobial resistance in shigella from Pakistan AND ( "2006/01/01"[PDat] : "2018/01/31"[PDat] )**  MeSH Terms: pakistan; anti infective agents; shigella  papx: anti infective agents |
| *Neisseria gonorrhoeae* | **antimicrobial resistance in N. gonorrhoeae in Pakistan**  MeSH Terms: anti infective agents; neisseria gonorrhoeae; pakistan  papx: anti infective agents |
| *S. aureus* | **community acquired methicillin-resistant *Staphylococcus aureus* in Pakistan**  MeSH Terms: pakistan; residence characteristics; methicillin resistant *Staphylococcus aureus*  **antimicrobial resistance in *Staphylococcus aureus* in Pakistan**  MeSH Terms: anti infective agents; pakistan; staphylococcus aureus, papx: anti infective agents |
| *S. pneumoniae* | **antimicrobial resistance in Streptococcus pneumoniae in Pakistan AND ( "2006/01/01"[PDat] : "2018/01/31"[PDat] )**  MeSH Terms: pakistan; anti infective agents; streptococcus pneumoniae  papx: anti infective agents |
